# Pattern of Malignancy in a Place in Western India

**DOI:** 10.1038/bjc.1970.79

**Published:** 1970-12

**Authors:** J. S. Kirtane, B. A. Sayed, V. P. Vaishnav

## Abstract

Analysis of 4500 cases of malignancy encountered in a general hospital in Western India showed that: (1) parts of buccopharynx were the site involved in nearly 25 per cent of these cases. In women the incidence of buccopharyngeal carcinoma was less than in men but was not insignificant (nearly 5 per cent of all malignancies found in women); (2) the cervix was the next frequent site involved accounting for 22 per cent of the total and 80 per cent of the female cancer; (3) cancer of breast was not less common (5 per cent of the total and 12 per cent of the female cancer); (4) oesophageal cancer was far more common than malignant neoplastic lesions of the other parts of the gastrointestinal tract (two-thirds of all cases being found in the oesophagus); (5) carcinoma of skin was not a common lesion.

High frequency of the types of cancer mentioned in the first two paragraphs is a “ommon facto” of many such reports from India. On the other hand, reported incidences of the types mentioned in the paragraphs number (3), (4) and (5) show wide variations in different parts of the country.


### PATTERN OF MALIGNANCY IN A PLACE IN WESTERN INDIA

J. S. KIRTANE, B. A. SAYED AND V. P. VAISHNAV

*From the Department of Pathology, Medical College, Baroda, India*

Received for publication October 1, 1970

**SUMMARY.—Analysis of 4500 cases of malignancy encountered in a general hospital in Western India showed that: (1) parts of buccopharynx were the site involved in nearly 25 per cent of these cases. In women the incidence of bucco-pharyngeal carcinoma was less than in men but was not insignificant (nearly 5 per cent of all malignancies found in women); (2) the cervix was the next frequent site involved accounting for 22 per cent of the total and 80 per cent of the female cancer; (3) cancer of breast was not less common (5 per cent of the total and 12 per cent of the female cancer); (4) oesophageal cancer was far more common than malignant neoplastic lesions of the other parts of the gastro-intestinal tract (two-thirds of all cases being found in the oesophagus); (5) carcinoma of skin was not a common lesion.**

**High frequency of the types of cancer mentioned in the first two paragraphs is a " common factor " of many such reports from India. On the other hand, reported incidences of the types mentioned in the paragraphs number (3), (4) and (5) show wide variations in different parts of the country.**

THIS communication is based on the records of Pathology Department, Medical College and Shri Sayajirao General (S.S.G.) Hospital, Baroda. It is likely to throw some light on the types of malignant neoplastic lesions encountered in this area.

### MATERIAL

Baroda is a rapidly industrialising city in Gujarat—a state in Western India. In the city of Baroda, S.S.G. Hospital is the only hospital equipped with modern amenities of diagnosis and treatment. There is no other similar hospital in the surrounding area of about 80 miles.

The city has a population of 600,000. The surrounding area is thickly populated (average 500 persons per square mile). The population is homogenous predominantly Hindu (91 per cent). Muslims are next in number (7·8 per cent). Christians are few (0·36 per cent).

All these three communities belong to the same ethnic group. Socially, they live the same way of life. The hospital is open to all communities irrespective of " caste ", creed or religion.

The present bed strength of the hospital is 900. On an average, 23,000 patients are admitted annually of whom about 10,000 are men, 9000 women and 4000 children.

Four thousand five hundred cases of cancer encountered during the 15-year period of 1952 to 1966 from amongst the patients of this hospital formed the basis of the present study. Cases diagnosed by haematological examination or cytology

(alone) are not included in this series. The number of specimens histologically examined during the period were 31,786.

RESULTS

On histopathological examination of 31,786 specimens, malignancy was diagnosed in 4500 cases. Thus the ratio of malignant neoplastic lesions to the total number of specimens examined came to 1 : 7. Most of these malignant tumours were epithelial. Sarcomas were few (280 cases or 6·2 per cent).

Distribution of malignancies according to the main anatomical sites involved is summarised in Table I.

TABLE I.—*Distribution of* 4500 *Cases of Cancer Detected at S.S.G. Hospital, Baroda* (1952–1966)

| Anatomic site | Number of cases | Percentage |
|---|---|---|
| Female genital tract | 1253 | 27·8 |
| Buccopharynx | 1128 | 25·0 |
| Larynx | 305 | 6·7 |
| Skin | 301 | 6·7 |
| G.I. tract including oesophagus | 270 | 6·0 |
| Male genital tract* | 249 | 5·5 |
| Breast† | 236 | 5·2 |
| Lymph nodes (metastatic) | 228 | 5·1 |
| Lymph nodes (primary tumours) | 109 | 2·4 |
| Bone (primary and secondary tumours) | 62 | 1·3 |
| All others (individually less than 1 per cent) | 274 | 6·0 |

* Constituted 9·7 per cent of male cancer. Cases of penile cancer were 157.
† Constituted 12 per cent of female cancer. Not a single case of male breast cancer was detected.

DISCUSSION

As mentioned before, our hospital being a general hospital strategically situated, the pattern of malignancy summarised in Table I may reflect the epidemiology of cancer in this area. It is clear without actually quoting figures from other authors for comparison that the reported prevalence of various types in Table I is in keeping with the general trend in India. In the paragraphs that follow only brief comments are offered on the first five categories listed in Table I.

*Female genital tract*

Cancer of the female genital tract accounting for 27·8 per cent of the total cancer (Table I) contributed 63·8 per cent of malignancies in women. In order of frequency the parts affected were cervix (1023 cases), uterus (86 cases), vagina (31 cases) and vulva (43 cases).

Cervical cancer was thus the most frequent variety of female cancer (52·4 per cent of all female cancer and 81·6 per cent of gynaecological cancer). The mean age of these patients was 41 years. High prevalence of cervical cancer and its occurrence at a relatively younger age in India are well known (Paymaster, 1964).

*Buccopharyngeal cancer*

In India, the incidence of buccopharyngeal cancer is said to be the highest in the world. The problem has attracted world attention (World Health Organisation, 1967).

In the present series, 39·5 per cent of all malignancies in men originated at these sites. In women, the incidence of buccopharyngeal cancer was much lower than in men but was not insignificant (4·8 per cent of all malignancies in women). The sites involved in the two sexes (as expected) were different.

In men the total number of cases was 1031. The oral cavity was involved in 146 cases (buccal mucosa in 38, anterior one third of tongue in 47, hard palate in 33 and lips in 28). The oropharynx was involved in 582 cases (posterior one third of tongue in 306, tonsils in 212 and soft palate in 64). The hypopharynx was the second most frequent site (303 cases).

In women only 97 cases were noted of which in about half (45 cases) the site involved was the hypopharynx. There were 26 cases each in the oral cavity and oropharynx.

The habit of chewing a mixture of tobacco and lime (with or without betel leaf) is often blamed for the higher incidence of oropharyngeal cancer in India and Ceylon (Sanghavi *et al.*, 1955) but there might be other factors at work (Tennekoon and Bartlett, 1969). This habit is very common in Gujarati men. Smoking of " bidis ", however, is more common—an almost universal habit in men.

*Larynx and skin*

Involvement of these two sites occurred with nearly equal frequency (Table I). The male : female ratio in skin cancer was 3 : 1. This was higher in laryngeal cancer, 8 : 1.

*Gastrointestinal tract including oesophagus*

As expected, this group consisted of 8·6 per cent and 2·6 per cent of the total male and female cancer respectively. Numerically oesophageal carcinoma was the most frequent subdivision accounting for 157 cases out of total 270.

Gastric carcinoma was the least frequent (0·6 per cent of the G.I. tract group). However, it may be of interest that four cases of what appeared to be primary Hodgkin's lymphoma of G.I. tract were detected during the period of study—three in the stomach and one in the transverse colon. One case of leiomyosarcoma and two of reticulum cell sarcoma involving stomach were also detected.

### REFERENCES

Paymaster, J. C.—(1964) *Cancer, N.Y.*, **17**, 1026.

Sanghavi, L. D., Rao, K. C. M. and Khanolkar, V. R.—(1955) *Br. med. J.*, i, 1111.

Tennekoon, G. E. and Bartlett, G.—(1969) *Br. J. Cancer*, **23**, 39.

World Health Organisation—(1967) ' Twenty Years in South East Asia 1948–1967 '. New Delhi (W.H.O. Regional Office for South East Asia).


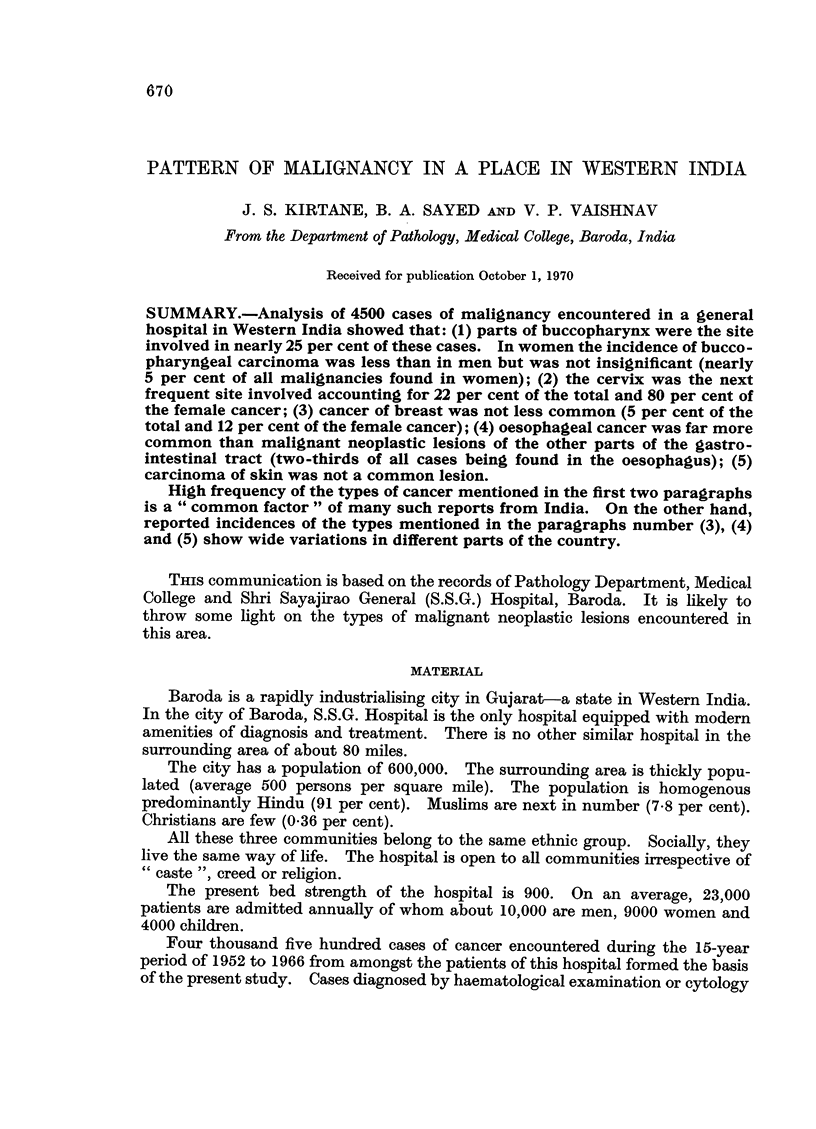

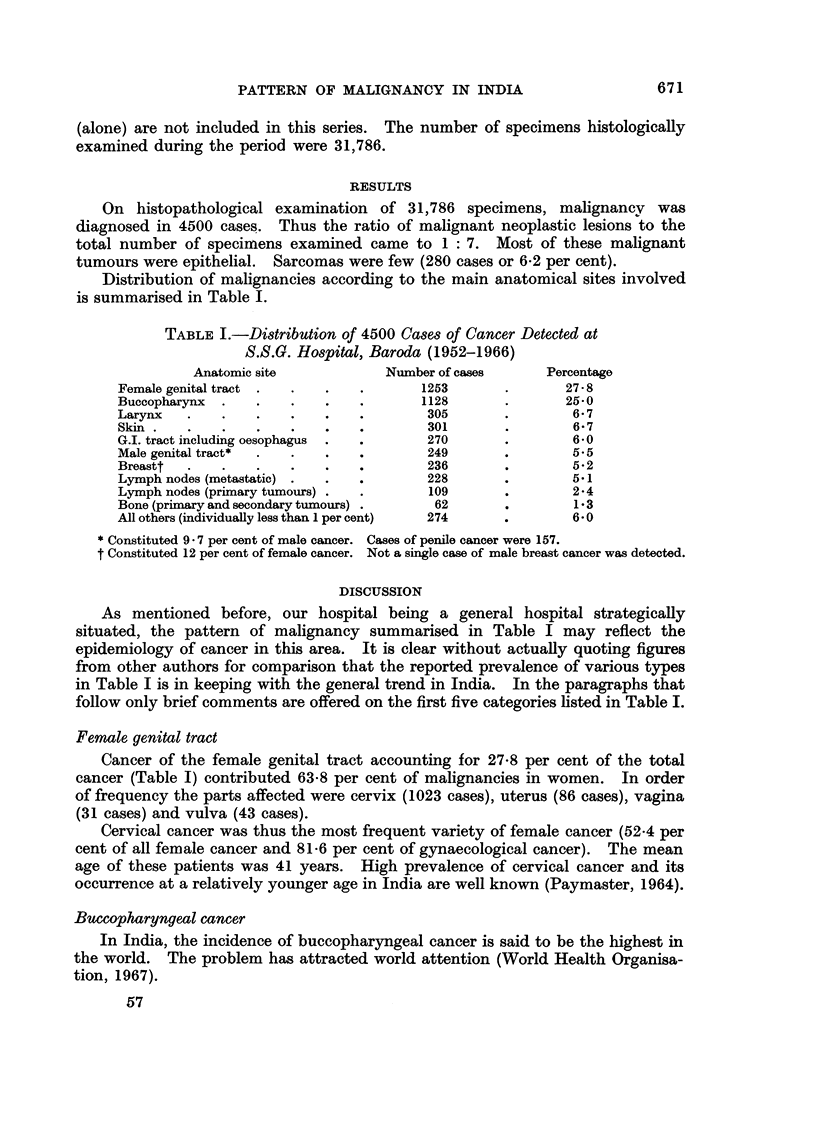

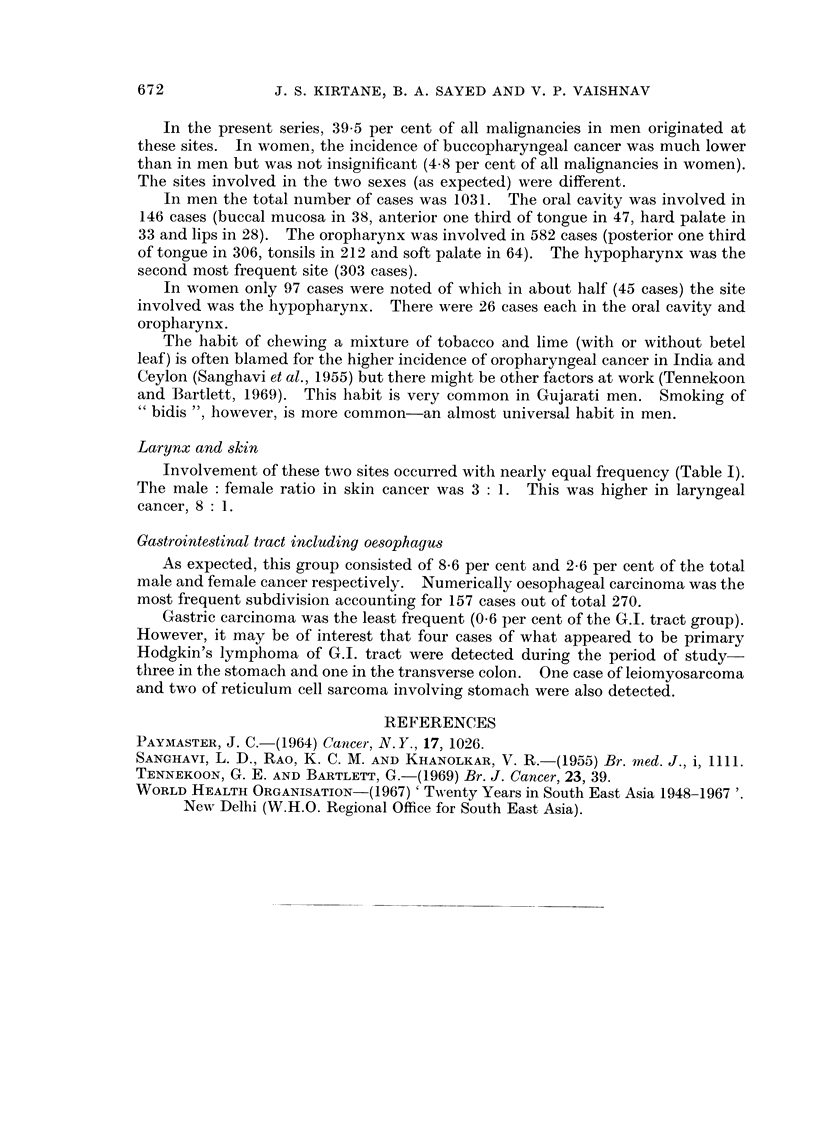


## References

[OCR_00164] PAYMASTER J. C. (1964). CANCER AND ITS DISTRIBUTION IN INDIA.. Cancer.

[OCR_00166] SANGHVI L. D., RAO K. C., KHANOLKAR V. R. (1955). Smoking and chewing of tobacco in relation to cancer of the upper alimentary tract.. Br Med J.

